# Combined Extract of *Leonurus japonicus* Houtt, *Eclipta prostrata* L., and *Pueraria lobata* Ohwi Improved Hot Flashes and Depression in an Ovariectomized Rat Model of Menopause

**DOI:** 10.3390/foods10010180

**Published:** 2021-01-18

**Authors:** Eun Young Kang, Hyun Kyung Kim, Ji Yeon Jung, Ji Hyun Kim, Tan Kyung Woo, Jeong In Choi, Jong Hoon Kim, Changwon Ahn, Hyeon Gyu Lee, Gwang-Woong Go

**Affiliations:** 1 Department of Food and Nutrition, Hanyang University, Seoul 04763, Korea; eunyoung94@hanyang.ac.kr (E.Y.K.); hyunkyung@hanyang.ac.kr (H.K.K.); 9404wd@gmail.com (J.Y.J.); jihy5686@naver.com (J.H.K.); tankwoo@naver.com (T.K.W.); 2 Research and Development Center, Nong Shim Co., Ltd., Seoul 07057, Korea; wjddls5651@nongshim.com (J.I.C.); kjh324@nongshim.com (J.H.K.); ahncw1@nongshim.com (C.A.); 3 Korean Living Science Research Center, Hanyang University, Seoul 04763, Korea

**Keywords:** *Eclipta prostrata* L., hot flashes, *Leonurus japonicus* Houtt, menopausal depression, *Pueraria lobata* Ohwi

## Abstract

Menopause leads to ovarian hormone loss, which causes symptoms such as weight gain, hot flashes, and depression. Exploring nutraceuticals is important for treating menopausal symptoms that extensively impact women’s quality of life. We hypothesized that a combination of *Leonurus japonicus* Houtt, *Eclipta prostrata* L., and *Pueraria lobata* Ohwi (LEPE) would alleviate menopausal symptoms in an ovariectomized menopausal rat model. Bilateral ovariectomy was performed and animals were assigned to five groups: (1) Sham, (2) Vehicle, (-) Control, (3) LEPE (100 mg/kg bw), (4) LEPE (200 mg/kg bw), and (5) Estradiol (3 μg/kg bw). LEPE was orally administered daily for 12 weeks. LEPE supplementation did not affect growth performance (body weight and feed intake) or body composition (lean mass and fat in tissue). LEPE did not cause deviations in aspartate aminotransferase, alanine aminotransferase, estradiol, and follicle-stimulating hormone levels, indicating no hepatotoxicity or endocrine disturbance. LEPE decreased type I collagen (CTX-1) but did not affect bone mineral density or osteocalcin. LEPE decreased tail temperature and increased rectal temperature, improving menopause-related vasomotor symptoms. Furthermore, LEPE ameliorated depression-related behavior, including in forced swimming and tail suspension tests. Thus, LEPE may improve menopausal symptoms by enhancing vasomotor symptoms and depression in an ovariectomized rat menopause model.

## 1. Introduction

Menopause refers to the permanent cessation of ovarian reproductive function and is divided into the perimenopausal, menopausal, and postmenopausal periods [1]. Perimenopause is also called menopausal transition, in which menstrual irregularities and estrogen decline occur. Menopause is diagnosed after amenorrhea for a full year and usually occurs in the late 40s to early 50s [2]. Natural menopause is a normal phenomenon that is part of aging. Menopause has various symptoms, such as hot flashes, night sweats, and vaginal dryness. Symptoms also include depression, nervous tension, palpitations, headaches, insomnia, lack of energy, difficulty concentrating, and dizzy spells called menopausal syndrome [3]. Menopause causes hormone levels to change, such as estrogen degradation, causing metabolic impairment [4]; that is, dyslipidemia, coronary heart disease, and osteoporosis. On average, menopause occurs at age 51 and the average lifespan is 80 years; thus, the average life expectancy after menopause is 30 years. This means that women spend approximately one-third of their lifetime postmenopausal. Therefore, the treatment of menopausal symptoms is critical to promote women’s health and improve their quality of life.

There are currently two main approaches to menopause treatments: hormone replacement therapy and non-hormonal therapy. Hormone replacement therapy includes therapy with a single hormone, such as estrogen or progestin, and a combination of estrogen and other medicines [5,6], which is an efficient treatment that dramatically relieves menopause-related symptoms. However, recent clinical studies have reported severe adverse effects, including the risk of carcinogenesis and cardiovascular disease [7,8]. Nonhormonal medication therapy, including selective serotonin reuptake inhibitors, and selective norepinephrine (NE) reuptake inhibitors, relieves hot flashes [6,9]. However, there are many side effects, such as headaches, excessive sweating, and constipation [10,11]. Therefore, nonhormonal therapy using natural bioactive compounds has emerged as an alternative. The global interest in complementary and alternative medicine is growing [12]. For this reason, it is imperative to study natural substances that have fewer side effects to relieve menopause symptoms.

*Leonurus japonicus* Houtt, also called motherwort, is used as a traditional medicinal herb in Asia and America [13]. *L. japonicus* Houtt has various benefits, such as anti-inflammatory, antioxidation, and antibacterial effects [14,15,16]. In particular, *L. japonicus* Houtt is traditionally used to treat menstrual irregularities, amenorrhea, and emmenagogue [17]. The bioactive components identified from *L. japonicus* include alkaloids, diterpenes, and flavones, especially leonurine and the stachydrine of alkaloids, have been widely investigated [18,19]. *Eclipta prostrata* L. is widely used in tropical and subtropical regions as tonics. *E. prostrata* L. is used traditionally for osteoblast differentiation and osteoclast degradation [20,21]. Numerous phytochemicals have been separated from *E. prostrata*, including triterpenes, flavonoids, thiophenes, coumestans, and steroids [22]. In particular, wedelactone, stigmasterol, β-terthienylmethanol were sufficiently studied in *E. prostrata* [23]. *Pueraria lobata* Ohwi, the root of kudzu, is used as an antidipsotropic and anti-diabetes agent as well as for the treatment of cardiovascular diseases in traditional Chinese medicine [24,25]. Puerarin, an isoflavone from *P. lobata* Ohwi, has been found to treat diabetes, dyslipidemia, neurotoxicity, and Alzheimer’s disease [26]. In addition, puerarin is a phytoestrogen that affects antidepressants and neurogenesis [27,28].

Combining the individual advantages of *L. japonicus* Houtt, *E. prostrata* L., and *P. lobata* Ohwi could create a synergic effect to relieve menopause symptoms. There have been no previous studies, to our knowledge, that have used the combination of these three herb extracts. Therefore, we hypothesized that the combined extracts of *L. japonicus* Houtt, *E. prostrata* L., and *P. lobata* Ohwi (LEPE), would mitigate menopause symptoms, including hot flashes, bone loss, and menopause-related depressive behavior, in ovariectomized (OVX) menopause rats without adverse effects.

## 2. Materials and Methods

### 2.1. Herbal Sample Preparation

LEPE, which is a combined extract of *L. japonicus* Houtt, *E. prostrata* L., and *P. lobata* Ohwi, was provided by Nong Shim Co. Ltd. (Seoul, Korea). LEPE was extracted with distilled water for 6–8 h at 95–120 °C. The extract was centrifuged at 7000 rpm for 10 min. Then, the supernatants were concentrated using a vacuum evaporator at 60 °C and spray-dried at 180–210 °C. Leonurine, wedelolactone, and puerarin were quantitatively analyzed as the standard substances of LEPE.

### 2.2. Animals and Diets

The Institutional Animal Care and Use Committee (IACUC) of Hanyang University approved all procedures of the animal experiment (HY-IACUC-19-0051). The animal experiment was conducted with 10-week-old female Sprague–Dawley rats. Each rat was housed in an individual cage and maintained under the following environmental conditions: 22 ± 1 °C, 12:12 h light–dark cycle, and 40–50% relative humidity. Food (PicoLab^®^ Rodent Diet 20 5053, Lab Supply, Fort Worth, TX, USA) and water were provided *ad libitum*. The rodent chow diet comprised 4.07 kcal/g (62% kcal from carbohydrate, 25% kcal from protein, and 13% kcal from fat).

After adaptation to a regular diet for one-week, bilateral ovariectomy was performed. The rats were anesthetized using a mixture of ketamine (100 mg/kg bw; Youhan Yanghang, Seoul, Korea) and xylazine (10 mg/kg bw; Bayer, Leverkusen, Germany). The sham group underwent surgery without tissue resection to provide the same stress as the other groups, and other rats underwent surgery to remove both ovaries. The rats had a week-long recovery period and the antibiotic Baytril 25 (Bayer) was used to prevent infection.

The rats were assigned to five groups (*n* = 12) as follows: (1) Sham, (2) Vehicle, a (-) control, (3) LEPE (100 mg/kg bw), (4) LEPE (200 mg/kg bw), and (5) estradiol (E_2_, estradiol 3 μg/kg bw), a comparative control. LEPE was dissolved in 10% Kolliphor^®^ EL (BASF, Ludwigshafen, Germany) and 0.9% saline solution and was orally gavaged daily for 12 weeks. The dosage of LEPE was determined based on a preliminary study (data not shown). In the preliminary study, LEPE decreased the tail temperature in OVX rats, indicating the alleviation of hot flashes, one of the symptoms of menopause. Estradiol was administered by subcutaneous injection every four days, depending on the hormone cycle.

### 2.3. Growth Performance and Body Composition

Body weight and feed intake were recorded weekly for 12 weeks. Fat in tissue, lean mass, and bone mineral density (BMD) was measured using dual-energy X-ray absorptiometry (DEXA, Medikors, Seoul, Korea). Rats were administered a mixture of ketamine and xylazine by intraperitoneal injection at the end of the experiment. Fat and lean mass were analyzed from cervical vertebrae 1 to the coccyx 4 bones. Bone density and mineral content, and the femur and tibia in the right legs, were analyzed.

### 2.4. Blood Biochemical Analysis

At the end of the animal experiment, blood was withdrawn via a cardiac puncture under anesthesia. Serum was separated by the concentration at 2000× *g* for 15 min at 4 °C and stored at −80 °C until further analysis. Aspartate aminotransferase (AST) and alanine aminotransferase (ALT) levels were determined using commercial kits (Asan Pharmaceutical, Seoul, Korea). Serum estradiol, follicle-stimulating hormone (FSH), and osteocalcin levels were tested using a rat ELISA kit (LSBio, Seattle, WA, USA). The C-terminal telopeptide of type I collagen (CTX-1) was determined using a rat CTX-1 ELISA kit (Biomatik, ON, Canada). Norepinephrine (NE) levels in serum were determined using a rat NE ELISA kit (MyBioSource, San Diego, CA, USA), and serotonin levels were measured using a rat ELISA kit (LDN, Nordhorn, Germany). Commercial assay kits were performed according to the manufacturer’s instructions.

### 2.5. Tail and Rectal Temperatures

The tail temperature was measured using an infrared thermometer (153-IRB, BiosebLab, Paris, France) 2 cm from where the tail meets the body of the rat. Rectal temperature was measured by inserting a probe (RET-2, Physitemp Instruments, Clifton, NJ, USA) at approximately 4 cm. During the temperature experiment, the laboratory was maintained at 22 ± 1.0 °C. The tail and rectal temperatures were recorded three times every week.

### 2.6. Behavior Analysis

*Tail suspension test (TST):* The TST was applied to assess menopause-related depression. In brief, rats were adjusted to the environment for 1 h. The rats were then suspended by tail adhesive tape to hang from a 50 cm height wherein one foot of the rat could reach the bottom. The observations were recorded and analyzed by dividing the agitating mobility and immobility. The results indicate that, as immobility increases, helplessness (an indicator of depression) becomes more severe.

*Forced swimming test (FST):* Menopause-related depression in OVX rats was evaluated using an FST. In brief, the OVX rats were placed in a cylindrical glass tank (height: 50 cm, width: 20 cm) filled with up to 30 cm of water to prevent the rats from touching the bottom and supporting themselves. The temperature of the water was adjusted to 25 ± 1 °C. The FST was conducted as a preliminary experiment, in which rats swam for 15 min a day before the main trial. The primary analysis involved forcing rats to swim for 5 min. All processes were recorded, and the FST results were analyzed by dividing climbing, swimming, and immobility. Increased climbing behavior indicated less helplessness, which indicated an improvement in depression symptoms.

*Elevated plus maze (EPM):* EPM was conducted to analyze anxiety among menopause-related depression symptoms. The apparatus was four-armed in the shape of a plus sign made of black acrylic. All arms were 10 cm in width × 50 cm in length and combined in the center to create a 10 cm^2^ center platform raised 50 cm above the floor. The two opposite arms were locked with walls, whereas the other two remained open without walls. Rats were allowed to move freely for 10 min at the beginning of the test. The test was recorded with a camera and the time spent in the closed and open arms was recorded. The longer the rats remained in a closed arm, the more anxious they felt.

### 2.7. Statistics Analysis

Data are presented as mean ± SEM (standard error of the mean). The results were analyzed using one-way ANOVA followed by Tukey’s multiple comparison test (Prism 6, GraphPad, La Jolla, CA, USA). A *p*-value < 0.05 was considered statistically significant.

## 3. Results and Discussion

### 3.1. Growth Performance and Body Composition

**Figure 1:**
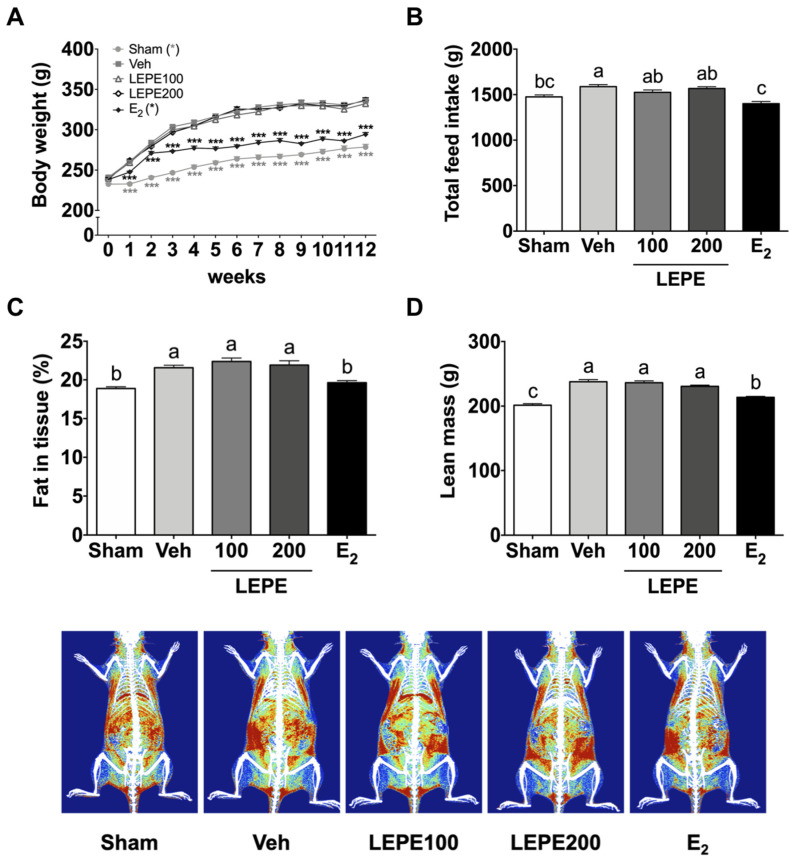
Growth performance and body composition in the ovariectomized rat model of menopause supplemented with LEPE. (**A**) Body weight, (**B**) total feed intake, (**C**) whole-body fat in tissue, and (**D**) lean mass. Sham, control sham-operated rats; Veh, ovariectomized (OVX) rats treated with vehicle; LEPE100, OVX rats treated with *Leonurus japonicus* Houtt, *Eclipta prostrata* L., and *Pueraria lobata* Ohwi extract (LEPE) 100 mg/kg bw; LEPE200, OVX rats treated with LEPE 200 mg/kg bw; E_2_, positive control, OVX rats injected with estradiol 3.0 µg/kg bw. Data are shown as mean ± SEM (*n* = 12). ^a–c^ Significant differences were analyzed by one-way ANOVA with Tukey’s multiple comparisons between groups (*p* < 0.05). *** *p* < 0.001 indicates a significant difference compared to the vehicle group.

Bilateral ovariectomy results in the absence of ovarian hormones in a rat model of menopause. Estrogen deficiency resulting from ovariectomy is responsible for increased feed intake and body weight gain, thereby increasing the fat in tissue via elevated levels of neuropeptide Y, an appetite stimulant, in the hypothalamus [29,30,31]. Growth performance and body composition were analyzed to confirm menopause induction in the experimental model by comparing the vehicle (negative control) and sham groups (Figure 1). Growth performance, including final body weight (121%) and total feed intake (108%) in the vehicle increased compared to the sham group (*p* < 0.001). In addition, in the vehicle, fat in tissue (114%) and lean mass (118%) significantly increased compared to the sham group (*p* < 0.001). These results indicate that OVX rat models of menopause were successfully established.

The functionality of LEPE was assessed in terms of growth performance and body composition (Figure 1). Neither LEPE100 nor LEPE200 altered body weight, feed intake, fat in tissue, or lean mass. Previously, no studies have combined LEPE, as in our study, to our knowledge. Instead, the results of prior studies of individual substances are as follows: the ethanol extract of *L. japonicus* (100 or 200 mg/kg bw) reduced the body weight and body fat of mice fed a high-fat diet (HFD) by downregulating hepatic lipogenesis and upregulating hepatic fatty acid oxidation [32]. The water extract (1, 10, or 100 mg/kg bw) of *L. sibiricus* L. (synonym with *L. japonicus*), containing leonurine as a bioactive compound, reduced body weight gain and fat accumulation by enhancing lipolysis in HFD fed OVX mice [33,34,35]. Aqueous *E. prostrata* leaf extract (1.4 g/kg bw) prevented body weight gain in OVX rats [36]. *P. lobata* ethanol extract (100 mg/kg bw) reduced body weight in OVX rats [37,38,39]. These results indicate that each of the three supplements had a body weight loss effect; however, LEPE did not. This could be because the mixed concentration of the three supplements that could have been effective was insufficient. Moreover, there may have been a difference in effect, based on whether the extracted solvent was in ethanol or aqueous form. Taken together, these results indicate that LEPE did not normalize increased body weight and fat mass induced by ovariectomy.

### 3.2. Evaluation of Hepatotoxicity and Endocrine Disturbance

The effects of LEPE on hepatotoxicity were estimated by measuring AST and ALT levels (Table 1). There was no significant difference between groups within the normal range (AST: 50–150 IU/L, ALT: 10–40 IU/L), indicating that LEPE did not adversely affect hepatotoxicity. No previous studies, to our knowledge, have reported toxicological results from LEPE treatment in vivo. Instead, the results of prior studies indicate the same trend with each individual component, which supports the results of the present study. For instance, the extract of *L. japonicus* (200 mg/kg bw) improved AST and ALT in HFD-induced obese mice [32]. Another study revealed that extracts of *Eclipta* species (500 mg/kg bw) improved AST and ALT in paracetamol-induced liver toxicity in rats [40]. In addition, the AST and ALT levels, which were elevated by the HFD, were notably augmented in the *Pueraria* root extract (300 mg/kg bw)-treated mice [41]. Toxicity studies of individual materials did not indicate problems with the liver function test, even at higher concentrations used in the present study. Therefore, LEPE is considered a safe functional nutraceutical that does not adversely affect liver function.

The serum levels of estradiol and FSH were measured to assess the effect on the endocrine system (Table 1). Estradiol is a pivotal estrogen steroid hormone that orchestrates female reproductive cycles and the endocrine system. The E_2_ group showed a considerable increase in estradiol concentration compared to the vehicle; however, LEPE groups did not affect estradiol. The pituitary gland releases FSH according to the menstrual cycle. FSH stimulates follicle development and estradiol production. The estradiol produces negative feedback to FSH [4,42]. According to the U.S. Food and Drug Administration (FDA), endocrine disruptor material interferes with the endocrine system, leading to adverse effects. Some chemicals disrupt the system by binding to receptors, such as estrogen receptors [43]. There was no significant difference in FSH level between groups. As a result, we estimated that LEPE did not cause any endocrine disturbance.

Uterine weight was determined to assess the effect of LEPE on the uterotrophic response (Table 1). According to OECD test guidelines, 440 compounds with estrogenic activity can cause endocrine disruption, resulting in uterine weight gain [44]. The vehicle showed that uterine tissue weight decreased by 80.4% compared to the sham group (*p* < 0.001). E_2_ increased the uterine weight by 257% compared to the vehicle; however, there was no difference in the LEPE groups. Similarly, estradiol treatment significantly increases uterine weight [45]. In summary, LEPE did not show adverse estrogenic activity, such as a uterotrophic response.

### 3.3. Osteoporosis

**Figure 2:**
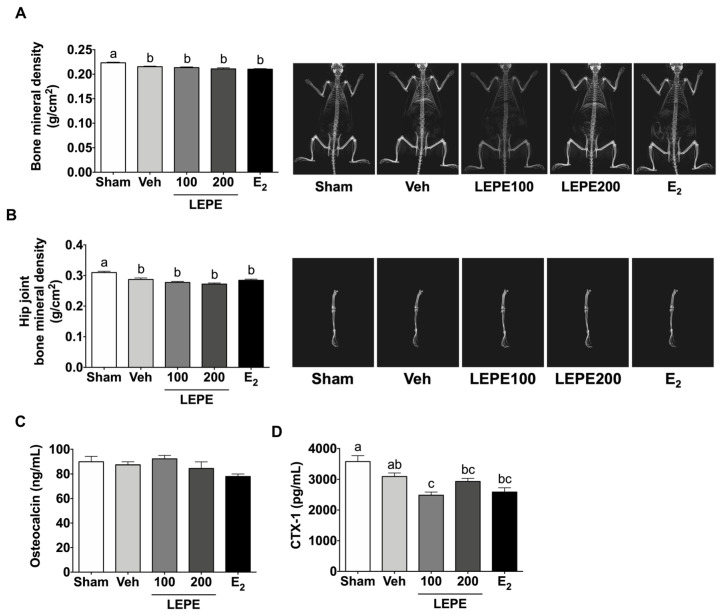
The effect of LEPE on menopause-related osteoporosis in body composition and blood. (**A**) BMD (bone mineral density), (**B**) hip joint BMD, (**C**) osteocalcin, and (**D**) CTX-1 (C-terminal telopeptide of type 1 collagen). Sham, control sham-operated rats; Veh, ovariectomized (OVX) rats treated with vehicle; LEPE100, OVX rats treated with *Leonurus japonicus* Houtt, *Eclipta prostrata* L., and *Pueraria lobata* Ohwi extract (LEPE) 100 mg/kg bw; LEPE200, OVX rats treated with LEPE 200 mg/kg bw; E_2_, positive control, OVX rats injected with estradiol 3.0 µg/kg bw. Data are shown as mean ± SEM (*n* = 12). ^a–c^ Significant differences were analyzed by one-way ANOVA with Tukey’s multiple comparisons between groups (*p* < 0.05).

In menopausal women, a lack of estrogen produces a higher risk of osteoporosis [46]. The effects of LEPE on osteoporosis were estimated by measuring whole-body BMD, hip joint BMD, osteocalcin levels, and CTX-1 (Figure 2). The vehicle showed lower whole-body (3.58%) and hip joint (7.30%) BMD than the sham group (*p* < 0.001). However, LEPE treatments showed no significant difference in whole-body and hip joint BMD. *L. sibiricus* L. (100 mg/kg bw) prevents the reduction in BMD in an LPS-induced osteoporosis mouse model [47]. The difference in these results is attributed to the different animal models of osteoporosis and different species in the Leonurus genus. Another study revealed that leonurine hydrochloride (15 mg/kg bw), a synthetic compound derived from *L. sibiricus*, increased BMD via intraperitoneal injection in OVX mice [48]. *E. prostrata* containing echinocystic acid increased BMD in OVX rats (5 or 15 mg/kg bw) [21]. As the reference studies applied a single bioactive compound, rather than a combined extract, these results were not consistent with the results of the present study. Aqueous *Ecliptae* herba (1.4 g/kg bw) prevented the reduction in BMD in OVX rats [36]. *P. lobata* vine ethanol extracts (20 mg/kg bw) suppressed the decrease in femoral BMD in OVX mice [38]. The main difference between prior studies and the present study is the use of individual extracts of higher concentrations compared to that of combined extracts. In addition, there is a solvent difference between ethanol and water. Therefore, contrary to expectations, LEPE did not restore BMD reduction in OVX rats.

Serum osteocalcin and CTX-1 are significant biomarkers for bone formation and absorption. Osteocalcin is formed in osteoblasts and accumulates in the extracellular matrix of the bone. By measuring osteocalcin levels, the degree of bone formation can be predicted. CTX-1 is useful as a predictor of fracture risk. First, the level of osteocalcin was not different in any group. CTX-1 in LEPE100 decreased by 19.7% compared to the vehicle (*p* < 0.001); however, LEPE200 did not alter CTX-1. The effects of LEPE have not previously been studied, to our knowledge. Instead, previous studies of extracts of the individual species have shown that *L. sibiricus* L. ethanol extract promoted osteoblast differentiation and suppressed osteoclast differentiation in vitro [47]. Wedelolactone, an *Ecliptae* herba compound, stimulates osteoblast differentiation, and bone mineralization in mouse bone marrow mesenchymal stem cells [49]. *E. prostrata* stimulates the proliferation of primary osteoblastic cells of newborn rats [20]. Ethanol extract levels of *P. lobata* Ohwi (100 mg/kg) decreased CTX-1 and osteocalcin in OVX rats, indicating bone absorption was reduced [37]. However, these results used ethanol solvent. In contrast, similar to our study, treatment with 1.4 g/kg aqueous *Ecliptae* herba for 12 weeks did not alter osteocalcin in OVX rats [36]. In summary, water-extracted LEPE supplementation was not effective in preventing menopausal osteoporosis.

### 3.4. Hot Flash Symptom Measured via the Tail and Rectal Temperatures

**Figure 3:**
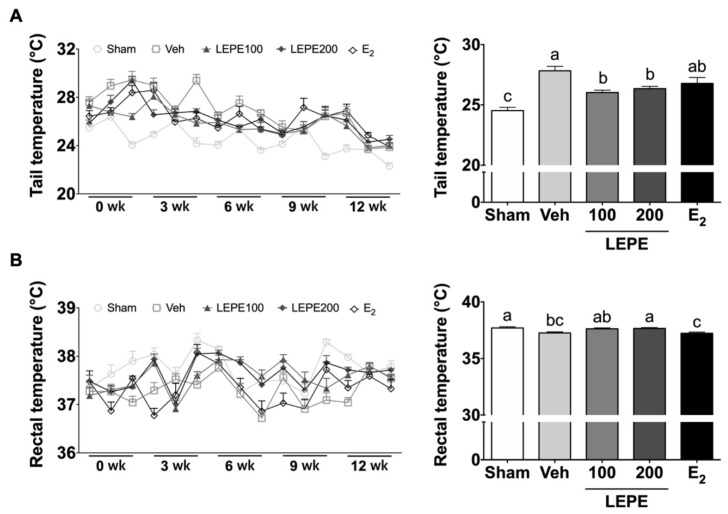
The effects of LEPE on hot flash-like responses via tail skin and rectal temperature measurements of the ovariectomized rat model. (**A**) Tail temperature and (**B**) rectal temperature. Sham, control sham-operated rats; Veh, ovariectomized (OVX) rats treated with vehicle; LEPE100, OVX rats treated with *Leonurus japonicus* Houtt, *Eclipta prostrata* L., and *Pueraria lobata* Ohwi extract (LEPE) 100 mg/kg bw; LEPE200, OVX rats treated with LEPE 200 mg/kg bw; E_2_, positive control, OVX rats injected with estradiol 3.0 µg/kg bw. Data are shown as mean ± SEM (*n* = 12). ^a–c^ Significant differences were analyzed by one-way ANOVA with Tukey’s multiple comparisons between groups (*p* < 0.05).

Hot flashes are a typical indication of vasomotor symptoms among menopausal women [50]. A lack of estradiol interrupts central thermoregulatory processing via interactions with the neurotransmitter system critical to temperature [51]. Tail and rectal temperatures represent the surface and core temperatures, respectively. The tail skin and rectal temperatures were determined three times a week at three-week intervals to assess whether LEPE treatment improved hot flashes (Figure 3). According to previous studies, the tail temperature shows a remarkable difference until seven weeks of post-ovarian resection operation, and rectal temperature shows a significant difference from eight weeks [52]. The tail temperature averages were calculated for three-to-six weeks. The tail temperature of the sham was 24.5 °C, and that of the vehicle was 27.8 °C, showing an increase of 3.3 °C (*p* < 0.001). According to a previous study of estradiol injection of 3 μg, the tail temperature decreased significantly from 10 days after OVX surgery [53]. Similarly, E_2_ showed that the tail temperature was 26.8 °C, a decrease of 1.0 °C compared to the vehicle. Strikingly, LEPE100 (26.0 °C) and LEPE200 (26.4 °C) showed a tail temperature decrease compared to the vehicle (*p* < 0.001).

The rectal temperature is presented as an average of six results from nine to twelve weeks. The rectal temperature of the sham was 37.7 °C and that of the vehicle was 37.3 °C, which showed a decrease of 0.4 °C (*p* < 0.001). In the preceding study, the increase in core temperature indicated a decrease in the surface temperature of the skin. The core temperature decreased when the surface temperature increased and *vice versa* [54]. LEPE200 increased a rectal temperature by 0.39 °C compared to the vehicle (*p* < 0.05). These findings are novel, as no previous study has demonstrated temperature improvement using LEPE, either individually or combined. Taken together, these findings indicate that vasomotor symptoms, such as hot flashes, could be improved by LEPE supplementation.

### 3.5. Effect of LEPE on Improving Menopause-Related Depression Based on Behavioral Tests and Blood Hormone Levels

**Figure 4:**
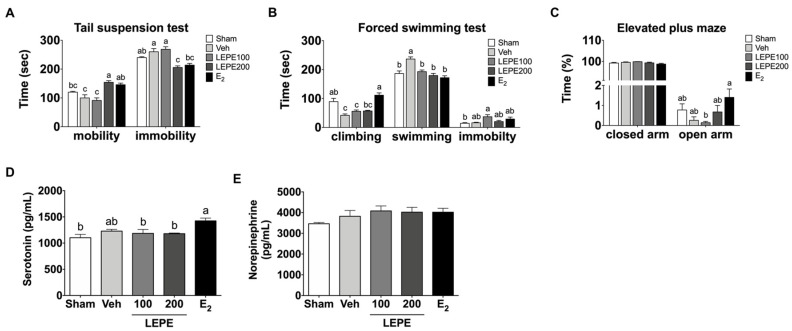
The effect of LEPE on improving menopause-related depression based on behavioral tests and blood hormone levels. (**A**) Time of mobility and immobility in the tail suspension test, (**B**) time spent climbing, swimming, and immobility in the forced swimming test, (**C**) time spent in the closed and open arms in the elevated plus-maze (**D**) serotonin and (**E**) norepinephrine levels in the serum. Sham, control sham-operated rats; Veh, ovariectomized (OVX) rats treated with vehicle; LEPE100, OVX rats treated with *Leonurus japonicus* Houtt, *Eclipta prostrata* L., and *Pueraria lobata* Ohwi extract (LEPE) 100 mg/kg bw; LEPE200, OVX rats treated with LEPE 200 mg/kg bw; E_2_, positive control, OVX rats injected with estradiol 3.0 µg/kg bw. Data are shown as mean ± SEM (*n* = 12). ^a–c^ Significant differences were analyzed by one-way ANOVA with Tukey’s multiple comparisons between groups (*p* < 0.05).

Depression is the most common behavioral comorbidity associated with menopause. In a cohort study, women who were followed up for eight years with no history of depression with hormonal changes after menopause often experienced the onset of depression [55]. Depressive behavior causes a lack of interest in pleasant activities as well as feelings of hopelessness. The TST and FST are widely accepted evaluating methods to test depression-like behavior in rodents [56,57]. The animal behavior analysis, including TST and FST, was performed to assess improvements in menopausal depression-like behavior (Figure 4A,B). The TST evaluated two elements: mobility and immobility for 360 s. As a result, the mobility in LEPE200 (155%) and E_2_ (146%) was higher than that in the vehicle (*p* < 0.001); however, LEPE100 did not alter mobility. In contrast, immobility in the LEPE200 (20.9%) and E_2_ (17.7%) was lower than that in the vehicle (*p* < 0.001). The FST analyzed three elements: climbing, immobility, and swimming for 300 s. The climbing in E_2_ increased by 266% compared to the vehicle (*p* < 0.001), and LEPE200 showed an increasing tendency of 136% compared to the vehicle. In addition, the swimming of LEPE100, LEPE200, and E_2_ decreased 18.4%, 24.1%, and 27.4%, respectively, compared to the vehicle (*p* < 0.001).

*Leonurus cardiaca* L. oil extract (1200 mg per day) improved anxiety and depression [58]. Leonurine (30 mg/kg) treatment decreased immobility in the TST and FST in chronic mild stress mice [59]. Puerarin (100 mg/kg/day) attenuated depression-like behavior in OVX mice by decreasing the immobility times of the TST and FST [28]. Puerarin decreased the immobility time of FST in chronic stress mice [60]. The EPM is used to assess anxiety-like behavior in rodents [61], as presented in Figure 4C. The EPM was measured for time spent in the closed and open arms for 600 s. The time spent in the open and closed arms of the OVX groups was not significantly different. There are no prior studies regarding the behavioral experiments of LEPE targeting EPM. Taken together, these findings suggest that LEPE supplementation could improve menopause depression in an OVX rat model.

Ovarian hormones regulate serotonergic and NE activity in the brain. Menopause-related estrogen deficiency reduces serotonin and NE levels in the brain [62,63]. Imbalance in the neurotransmitter systems of serotonin and NE can lead to depression, anxiety, and excess anger. Serotonin and NE, pivotal neurotransmitters, were measured in serum to evaluate menopause-related depression (Figure 4D,E). They showed no significant differences in any OVX rats. A previous study reported that leonurine increased serotonin and NE levels in the hippocampus and prefrontal cortex of chronic mild stress mice [59]. The results of this study differ from those of the present study. There is no sufficient evidence to indicate that LEPE recovered serotonin and NE caused by an ovarian hormone deficiency.

## 4. Conclusions

LEPE did not affect the growth performance (body weight and feed intake) or body composition (fat mass and lean mass) in OVX rats. LEPE did not cause changes in AST, ALT, estradiol, or FSH levels, indicating no hepatotoxicity or endocrine disturbance. Among the biomarkers for osteoporosis, LEPE reduced CTX-1; however, BMD and osteocalcin did not change. Strikingly, LEPE decreased tail temperature, indicating improved vasomotor symptoms, such as hot flashes. Furthermore, LEPE ameliorated depression-related behavior, including TST and FST. However, the scope of this study was limited to identify the phenotype recovery of menopausal symptoms by LEPE supplementation. Further studies are required to explore the biomolecular mechanism of improving vasomotor symptoms and depressive related behavior. In conclusion, our findings suggest that LEPE may relieve menopause symptoms by improving vasomotor symptoms and depression in an OVX rat menopause model.

## Figures and Tables

**Table 1:** Evaluation of hepatotoxicity and safety of LEPE in serum.

	Sham ^2^	Vehicle	LEPE100	LEPE200	E_2_
AST (IU/L) ^1^	107.50 ± 10.14	108.20 ± 7.98	130.40 ± 4.90	114.90 ± 6.43	83.05 ± 17.11
ALT (IU/L)	33.42 ± 2.33	41.70 ± 5.10	39.35 ± 5.53	37.75 ± 3.70	41.03 ± 4.01
Estradiol (pg/mL)	440.90 ± 22.85 ^ab^	408.60 ± 8.79 ^b^	456.10 ± 14.11 ^ab^	460.30 ± 13.93 ^ab^	479.70 ± 11.92 ^a^
FSH (ng/mL)	1.88 ± 0.36	1.09 ± 0.26	0.66 ± 0.05	1.63 ± 0.29	1.66 ± 0.29
Uterine weight (mg)	602.60 ± 42.79 ^a^	118.10 ± 7.50 ^c^	117.30 ± 6.94 ^c^	118.00 ± 3.53 ^c^	303.60 ± 14.26 ^b^

^1^ AST, aspartate transaminase; ALT, alanine aminotransferase; FSH, follicle-stimulating hormone. ^2^ Sham, sham-operated rats; Vehicle, ovariectomized (OVX) rats treated with vehicle; LEPE100, OVX rats treated with *Leonurus japonicus* Houtt, *Eclipta prostrata* L., and *Pueraria lobata* Ohwi (LEPE) 100 mg/kg bw; LEPE200, OVX rats treated with LEPE 200 mg/kg bw; E_2_, positive control, OVX rats injected with estradiol 3.0 µg/kg bw. Data are shown as the mean ± SEM (*n* = 12). ^a–c^ Significant differences were analyzed by one-way ANOVA with Tukey’s multiple comparisons between groups (*p* < 0.05).

## Data Availability

The data presented in this study are available on request from the corresponding author.
